# The Danish Version of the Problem Areas in Diabetes-Teen (PAID-T) Scale: Translation and Linguistic Validation

**DOI:** 10.1155/2023/4655563

**Published:** 2023-06-23

**Authors:** Marie Ørts Rahbæk, Sissel Due Jensen, Karina Kudahl Hansen, Annelli Sandbæk, Sten Lund, Anette Andersen

**Affiliations:** ^1^Steno Diabetes Center Aarhus, Aarhus University Hospital, Aarhus, Denmark; ^2^Department of Public Health, Aarhus University, Aarhus, Denmark

## Abstract

**Introduction:**

Diabetes distress is often seen in adolescents with Type 1 diabetes (T1D). Problem Areas in Diabetes (PAID) is the most frequently used scale to assess diabetes distress in clinical settings, but the version for teenagers has not been translated into Danish and validated before now.

**Objective:**

This study describes the translation into Danish of the PAID-T scale, which was developed to measure emotional distress in teenagers with diabetes.

**Materials and Methods:**

The study was conducted in two phases. First, the PAID-T was translated into Danish based on the guidelines from the International Society for Pharmacoeconomics and Outcome Research and a forwardbackward translation procedure. Second, cognitive interviews were conducted, and the Danish version of the PAID-T was modified to ensure linguistic equivalence with the original scale in English.

**Results:**

The Danish version of the PAID-T questionnaire was found to be understandable and relevant for adolescents with T1D. No questions were found to be irrelevant. However, the cognitive interviews showed that the issue of balancing alcohol intake and blood sugar levels was not covered by PAID-T, although this was found relevant in the Danish target group.

**Conclusion:**

This study described the translation and linguistic validation of the PAID-T scale into Danish. After modifications based on the feedback from the cognitive interviews, the Danish version was found to be linguistically equivalent to the original English version.

## 1. Introduction

Diabetes-related distress is commonly seen in adolescents. Around one-third of young people diagnosed with diabetes experience diabetes-related distress [1, 2]. Managing diabetes in everyday life is a demanding task in adolescence. It requires a balance of taking care of the chronic disease and incorporating competing interests in adolescence, such as peer relationships, romantic relations, educational tasks, and engaging in risk behavior. It is well-known that diabetes management in adolescence is often characterized by fluctuating glycaemic variability, absence from clinical attendance, and poor self-care [3, 4]. This could explain why many diabetes-related factors may invoke negative feelings or distress among teenagers with diabetes [2]. Few studies have investigated diabetes-related distress in an adult population in Denmark, such as the relationships between work-related factors, diabetes distress, intentional hyperglycemia at work, and glycaemic variability [5]. Another study among emerging adults aged 18–30 years with Type 1 diabetes (T1D) in 17 countries, one of them being Denmark, suggests that emerging adults are less engaged in self-management and experienced greater diabetes-related distress [6]. For children and adolescents with T1D aged 8–18, DISABKIDS measuring health-related quality of life has been translated and validated in Danish [7]. Nevertheless, diabetes-related distress specifically has not been examined systematically among adolescents in Denmark.

In the clinical setting, it is important to be able to detect early signs of diabetes distress in adolescents to facilitate early intervention with a view to mitigating these feelings and reducing negative development. A validated instrument to assess the emotional status of teenagers with diabetes would be useful for healthcare professionals in contact with these teenagers and would also be a valuable tool for researchers in Denmark, where until now no validated instrument for assessing diabetes-related distress have been translated into Danish.

The Problem Areas in Diabetes (PAID) scale was originally developed for (and validated in) an adult population [8]. However, two adapted versions have since been made for younger populations; PAID-C for children aged 8–12 years [9] and PAID-T for teenagers aged 13–19 years [10], as well as versions for parents of children aged 8–12 years, P-PAID-C [11], and parents of teenagers, P-PAID-T [12]. Recently, a validated version for emerging adults aged 18–30 years, PAID-EA, has also been developed [13]. A review from 2015 on instruments measuring diabetes-related emotional distress in adults concluded that PAID was the most frequently used and the best validated of these instruments [14]. Previous validations of the PAID-T scale have shown good face validity and strong internal consistency and reliability [10, 12]. The PAID-T scale has been translated into several languages, but not into Danish until now.

The aim of this study was to translate and linguistically validate the English version of the PAID-T into Danish for use in a clinical setting and for research purposes.

## 2. Materials and Methods

The study was conducted in two phases: translation and validation of the PAID-T (Phase 1) and cognitive interviews (Phase 2). Phase 1 was conducted by a steering group, whereas Phase 2 was conducted by two experienced qualitative researchers forming part of the steering group.

### 2.1. Steering Group

The process of translation and validation was conducted by a steering group. Group members were carefully selected to ensure complimentary skills. SL (doctor) and KKH (nurse) were both employed in an outpatient clinic for adolescents with T1D and were both familiar with the target group. AS has worked as a researcher in academia for many years, with a special focus on general medicine and diabetes. AA has extensive experience with validated scales. MØR and SDJ are both professionally trained qualitative researchers with special knowledge of living with diabetes. AS, AA, and MØR participated with the translator in harmonization between the different versions. SL and KKH gave feedback from a clinical perspective based on their experience with the target group, and finally SDJ and MØR conducted the cognitive interviews.

### 2.2. *Translation Procedure* (*Phase 1*)

**Figure 1:**
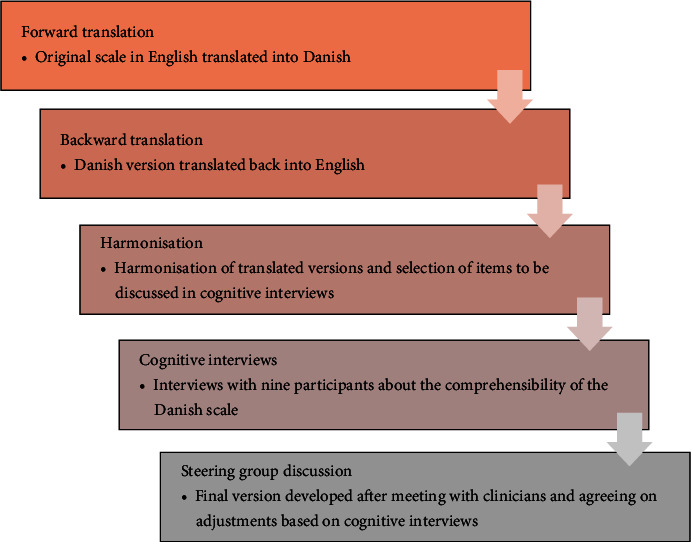
Steps in the translation approach.

The Danish translation and linguistic validation of PAID-T was guided by the protocol and guidelines from the International Society for Pharmacoeconomics and Outcome Research (ISPOR) [15]. The translation was produced by a forward–backward procedure (see Figure 1), which is a widely used and generally acknowledged approach in health care [16].

A native Danish speaker with academic English experience initially translated the English PAID-T scale into Danish (forward translation). The forward translator consulted the adult version of PAID, which had previously been translated into Danish; two items are identical between the English versions of PAID and PAID-T, and the wordings are very similar in several other items of these two questionnaires. The Danish forward translation was then translated back into English (back translation) by an American who has lived in Denmark for many years and works as a professional translator. The back translator received a translation brief on the purpose and target audience; it also stated that emphasis should be placed on conceptual and cultural equivalence. To ensure conceptual equivalence, the original PAID-T and the two translations (forward and backward) were assembled into one table, making it possible to see any discrepancies between the three versions for each item. Any divergences were marked in red font and harmonized through discussions in the steering group.

Twelve items were selected for cognitive interviewing because the steering group was uncertain if the target group (teenagers) would comprehend the intended meaning or whether it correlated with the language of the clinical staff (i.e., how issues are framed in the clinical setting).

### 2.3. *Cognitive Interviews* (*Phase 2*)

Three inclusion criteria were used to identify eligible participants: (1) a diagnosis of T1D, (2) age of 13–19 years, and (3) ability to speak and read Danish. In the recruitment process, all eligible patients with a booked appointment in the clinic within a week were invited to participate by a nurse. As this resulted in not all ages being covered, the nurse would subsequently invite patients who matched the ages missing from the sample. Thirteen participants were invited for interviews, of which nine consented and agreed to participate. The four participants who declined participation were all boys aged 13 years, who declined due to lack of interest or their parents' concern whether filling in the questionnaire and participating in the interview would have a negative influence on their diabetes management and self-care. One mother expressed, that the items were all worded negatively or discouraging, and therefore she was not interested in him participating.

Nine adolescents with T1D were included for cognitive interviews about the Danish translation of PAID-T to assess cognitive equivalence. The sample size was decided based on the recommended number of participants specified in the ISPOR guidelines [15]. The nine participants (six females and three males) were between 14 and 17 years of age and all of Danish origin (see Table 1). The cognitive interviews lasted between 15 and 40 min each and took place either by phone or in-person attendance at a diabetes-outpatient clinic.

Cognitive validation was performed through individual semistructured interviews (held by MØR and SDJ) and the use of a respondent debriefing methodology. In this technique, the interviewer probes for specific information after completion of the questionnaire by the respondent, e.g. what difficulties were experienced while completing the items, and how the respondent came to choose their answers. This way, information was elicited on the clarity of instructions, response categories, and overall comments on the difficulty and relevance of the scale [17].

Before the interview started, the participants were individually introduced to the PAID-T questionnaire and the overall aim, use, and storage of the interview. The interviewer then encouraged the participants to give their honest and straightforward opinion on how they experienced the language and comprehensibility of the PAID-T questionnaire. They were then asked to fill in the questionnaire on their own and mark any words or expressions that were unclear or they felt uncomfortable with. The participants' understanding and interpretation of the introductory instructions and the 26 items were then systematically reviewed, and the participants were asked whether each of their marked items were difficult to understand, confusing, or difficult to answer. Based on the harmonization discussion in the steering group on ambiguous wording, the participants were specifically asked about the meaning and interpretation of 12 items. One example was whether they preferred the Danish word *komplikationer* or *senfølger* (in English “complications”) when describing their worries for future severe complications in Item 6. If the participants indicated that the wording or meaning was unclear or ambiguous, they were asked to phrase the question in their own words. In closing, the participants were asked about their general thoughts on answering the questionnaire, how they experienced the response categories, if there were topics relevant to their experiences of emotional distress related to diabetes that were not already covered in the scale.

### 2.4. Analysis

The interviewers made notes throughout the interviews. After each session, a recording of the interview was transcribed and condensed. All comments from the participants were gathered into a table with respondents in columns and items in rows to give a complete overview of the total interview material. Through a content analysis, the participants' overall understanding of the questionnaire and emphasized items as well as other themes relevant for their experiences were identified [18]. These insights were discussed within the steering group.

## 3. Results

### 3.1. Forward and Backward Translation

In total, 13 items out of 26 had markings in red font to indicate discrepancies between the original English version and the back-translation; only one was an entire sentence, and the remaining 12 were single words or phrases that had been altered through translation. None of these had significantly changed the meaning of the original version. One example was Item 7: *Feeling upset when my diabetes gets* “*off track*” became *I get upset when my diabetes gets* “*out of control*” in the back-translation. Through a harmonization process, the steering group discussed each of these divergences and decided on a final version of the scale to be used in the cognitive interviews. In 12 items, ambiguous or difficult words or phrases were selected to be specifically explored in cognitive interviews, which will be further described in the following section.

### 3.2. Cognitive Interviews

Nine adolescents participated in the cognitive interviews. Eight had read the introductory instructions. However, four indicated that they would most likely have skipped the introduction if they were to complete a similar questionnaire in another setting.

Overall, the participants found the PAID-T questionnaire understandable and relevant according to their experience with having T1D. None of the participants found any of the questions uncomfortable, and none of them received questions that they did not want to answer. The interviews elicited 16 total comments on 13 different items. Five comments (on four different items/questions) came from five different participants, whereas the remaining 11 comments were prompted on the basis of the interests of the project group. When asked if any aspects of living with diabetes as a teenager were missing in the questionnaire, two 17-year-old mentioned balancing of alcohol and blood sugar levels.  Table 2 summarizes the comments made on each item by the participants.

### 3.3. Linguistic Modifications

After completion of interviews, the steering group met to discuss the results from the nine interviews and determined that changes were required in seven items. As shown in Table 2, these changes were based on comments made by the participants on comprehensibility and terms that better suited their experiences of diabetes management and ways of expressing it. One example was the term “*complications*” in Items 6 and 26, which refers to possible future illnesses caused by diabetes. The direct Danish translation is “*komplikationer*”, while “*følgesygdomme*” and “*senfølger*” are other terms for the same. Through the interviews, it became clear that “*følgesygdomme*” or “*senfølger*” was preferred over “*komplikationer*” by the participants to describe this risk. Therefore, the steering group changed the wording into “*følgesygdomme*”, which is used as a medical term and in the patients' everyday language as well.

The linguistic changes were deemed necessary to make the questionnaire linguistically appropriate for the target group, while still maintaining the inherent meaning of the original questions. See Table 2 for an extensive list of decisions made by the steering group.

## 4. Discussion

This paper outlines the process of translating and linguistically validating a Danish version of the PAID-T instrument. This process was guided by the ISPOR guidelines [15]. The end result was a conceptually equivalent and cross-culturally adapted version. Cognitive interviews elicited 16 comments on 13 items. Based on these comments, modifications were required in seven items to enhance the comprehensibility to adolescents, while maintaining the original meaning of the items.

The participants stated that the questionnaire was relevant and captured their experiences of living with T1D. Still, few comments were made on topics that were not included; alcohol was mentioned as a relevant topic for living with diabetes as a teenager. As the original questionnaire was developed in the US, where the minimum legal age of drinking (and the age of debut of drinking) is higher than in a Scandinavian setting, issues of balancing T1D with alcohol intake may not have been relevant to adolescents in the US. However, in Denmark, young people aged 16–18 years are legally allowed to buy products containing less than 16.5% alcohol. Atleast 34% of girls and 39% of boys aged 13 years have experience with drinking alcohol, although only 2% report weekly consumption [19]. Therefore, an item regarding alcohol could be relevant to the target group (adolescents) in a Danish setting. This raises an interesting point on the cultural dimensions of validated questionnaires across countries and cultures. The recently developed PAID-EA, which was validated in as US setting among emerging adults aged 18–30, does include alcohol-consumption in Item 6: “*I worry about being able to socialize because of how alcohol affects my blood sugar*” [13]. Given the cultural context in Denmark as outlined above, it is worth considering if a Danish diabetes measure should include a similar item for older teenagers to open a conversation with their care providers in the clinic.

The four teenagers who declined participation were all boys aged 13, mostly due to a lack of interest. But it was interesting that some parents were concerned that filling out the questionnaire and participating in the interview would cause a negative influence on their child's diabetes management. The concern was based on the negative or discouraging formulation of the items. Future research exploring how the teenagers are affected by answering the PAID-T and if age or gender plays a role could be interesting to pursue.

An important strength was that the translation process followed an acknowledged guideline. An additional strength was the application of a thorough cognitive interviewing methodology, with interviews being recorded and transcribed for accuracy. Our sample size was just above the recommended 5–8 patients. Yet, it must be noted that the cognitive interviews were conducted only with patients from Aarhus University Hospital in the Central Denmark Region. This group consisted of more girls than boys, all of Danish ancestry, and no one above the age of 17 or below 14 years. Consequently, our sample does not reflect the exact proportions of the target population of the questionnaire [2, 10]. However, the large number of participants combined with the thorough cognitive interviewing process and the linguistic modifications ensured that the final translation was linguistically appropriate for adolescents.

Teenagers may talk about diabetes in their daily life in a different way than diabetes is articulated in a hospital setting. Through cognitive interviews, we were able to explore whether the translated words and phrases were comprehensible and relevant for teenagers, and we also took into account their feedback when making the final Danish translation.

The guidelines recommend having two forward and two backward translators. As a Danish version of PAID for adults already existed (with two items being identical to PAID-T and several other overlapping phrases), only one forward and backward translation was deemed adequate. The end result was found to be consistent with the English language version.

## 5. Conclusion

This study describes the process of translating and linguistically validating PAID-T from English into Danish. Cognitive interviews with nine participants resulted in modifications, and the results suggest that the Danish translation is linguistically equivalent to the English version.

The Danish version of the PAID-T is already used as a screening tool in outpatient clinic visits for adolescents with T1D at Aarhus University Hospital. The next steps could include further validation of the PAID-T in the population, e.g., examination of the construct validity by using Rasch models and assessing differential item functioning. This will help us understand whether differences exist between the scores of boys and girls, different age groups, ethnicities, etc.

## Figures and Tables

**Table 1:** Characteristics of participants.

No.	Age	Sex	Ethnic origin
1	14	Female	Danish
2	15	Female	Danish
3	16	Male	Danish
4	16	Female	Danish
5	17	Female	Danish
6	17	Male	Danish
7	17	Male	Danish
8	17	Female	Danish
9	17	Female	Danish

**Table 2:** Overview of comments from participants and changes made by the steering group.

Item	Comments from participants	Comments from steering group	Suggested wording from participants	Decision by steering group
2	Needed to be read twice to understand the wording “Not knowing if” (4).			

3		The Danish wording “fylde for meget” (feeling overwhelmed) may hold both an emotional and a time dimension.	The majority of participants understood the phrase “fylde for meget” as in “thinking a lot about it”.	

3 and 18	The wording of “min diabetesbehandling” (diabetes regimen) was unclear for two participants, who asked whether “min diabetesbehandling” referred to the isolated activity of taking insulin or going to the hospital, or whether “min diabetesbehandling” referred to the everyday task of taking care of their diabetes disease (8 and 9).	It was unclear whether the statement “min diabetesbehandling” refers to taking medicine/going to the hospital, or if it refers to the everyday task of managing the diabetes care.	They suggested “min daglige håndtering af diabetes” (my daily diabetes management) instead of “min diabetesbehandling”.	Changes are required. From “min diabetesbehandling” to “min daglige håndtering af diabetes”.

6 and 26		The term “komplikationer” (complications) may refer to a language used in medical settings, whereas “senfølger” or “følgesygdomme” is a term closer to their everyday language.	Six out of nine participants preferred the term “senfølger” or “følgesygdomme” to “komplikationer”.	Changes are required. From “komplikationer/følgesygdomme” to “følgesygdomme”

7		Additionally, using the Danish terms “berørt” or “påvirket” for upset made most sense to the participants.	All participants agreed on the use of the term “påvirket”; several argued that “berørt” was mostly related to being sad, while “påvirket” encompassed a greater variety of feelings or reactions, such as anger, frustration, and discouragement.	

7		It was unclear whether the translation of the metaphor “løber af sporet” (get off track) made sense and was understandable to the participants.	All participants agreed that this metaphor described their experiences of losing control of their blood sugar level, i.e., when too high/low.	

8	The term “udbrændt” was difficult to understand and needed to be explained to one participant (1).	Did the Danish term “udbrændt” (burned-out) correspond to the teenagers' experiences of diabetes-related emotional distress? Was it comprehensible?	Several participants were unsure of the meaning of the term “udbrændt” and instead suggested the terms “udmattet” (exhausted) or “udkørt” (worn out).	Changes are required. From “udbrændt” to “udkørt”

11		Did the Danish wording “tage mig af min diabetes” (keep up with my daily diabetes tasks) make sense to the teenagers?	The majority suggested to change the wording “tage mig af” (keep up with) to “passe min” (take care of).	Changes are required. From “tage mig af” to “passe min”

12	Two participants highlighted the term “modløs” (discouraged) as difficult to understand, but they did understand the question due to the following term “opgivende” (defeated) (6 and 9).			

12		It was unclear whether the translated term “måleapparat” (meter) made sense to the participants.	All participants agreed that “måleapparat” was the most accurate term.	

13		It was unclear whether the translated metaphor “diabetespolitiet” (diabetes police) made sense to the participants.	All participants understood the metaphor in the sense of wanting to control and keeping an eye on their diabetes management.	

15		Was the term meaningful to the participants?	All but one understood the wording as a perfection in their effort to manage their diabetes as in a dead straight blood sugar level at 6. Only one understood the question as if *he* had to be perfect to manage his diabetes.	

16		It was unclear whether the translated terms “glemmer” or “dropper” (missing) made most sense to the participants.	All but one agreed that “glemmer” was the most accurate word since “dropper” refers to conscious action, which is represented in the following word “springer over” (skipping).	

17		It was unclear whether the translated terms “vildt” or “meget” (wildly) made most sense for the participants.	All participants agreed that “meget” was the most accurate word.	

24		It was unclear whether the meaning of the item would be clearer if “uden for hjemmet” (outside of home) was added.	The participants agreed that adding “uden for hjemmet” would clarify the meaning of the question.	Changes are required. Add “uden for hjemmet”

Numbers in parentheses refer to the participant making the comment.

## Data Availability

Access to data is restricted to the public research community due to ethical concerns of patient privacy and confidentiality.
